# Sex Chromosome Evolution: So Many Exceptions to the Rules

**DOI:** 10.1093/gbe/evaa081

**Published:** 2020-04-21

**Authors:** Benjamin L S Furman, David C H Metzger, Iulia Darolti, Alison E Wright, Benjamin A Sandkam, Pedro Almeida, Jacelyn J Shu, Judith E Mank

**Affiliations:** e1 Beaty Biodiversity Research Centre, University of British Columbia, Vancouver, British Columbia, Canada; e2 Department of Zoology, University of British Columbia, Vancouver, British Columbia, Canada; e3 Department of Animal and Plant Sciences, University of Sheffield, United Kingdom; e4 Department of Genetics, Evolution and Environment, University College London, United Kingdom

**Keywords:** recombination suppression, sex determination, inversions, methylation, dosage compensation

## Abstract

Genomic analysis of many nonmodel species has uncovered an incredible diversity of sex chromosome systems, making it possible to empirically test the rich body of evolutionary theory that describes each stage of sex chromosome evolution. Classic theory predicts that sex chromosomes originate from a pair of homologous autosomes and recombination between them is suppressed via inversions to resolve sexual conflict. The resulting degradation of the Y chromosome gene content creates the need for dosage compensation in the heterogametic sex. Sex chromosome theory also implies a linear process, starting from sex chromosome origin and progressing to heteromorphism. Despite many convergent genomic patterns exhibited by independently evolved sex chromosome systems, and many case studies supporting these theoretical predictions, emerging data provide numerous interesting exceptions to these long-standing theories, and suggest that the remarkable diversity of sex chromosomes is matched by a similar diversity in their evolution. For example, it is clear that sex chromosome pairs are not always derived from homologous autosomes. In addition, both the cause and the mechanism of recombination suppression between sex chromosome pairs remain unclear, and it may be that the spread of recombination suppression is a more gradual process than previously thought. It is also clear that dosage compensation can be achieved in many ways, and displays a range of efficacy in different systems. Finally, the remarkable turnover of sex chromosomes in many systems, as well as variation in the rate of sex chromosome divergence, suggest that assumptions about the inevitable linearity of sex chromosome evolution are not always empirically supported, and the drivers of the birth–death cycle of sex chromosome evolution remain to be elucidated. Here, we concentrate on how the diversity in sex chromosomes across taxa highlights an equal diversity in each stage of sex chromosome evolution.

## Introduction

The presence of separate sexes is found throughout the tree of life, and is particularly prevalent in metazoans. When present, the development of separate sexes requires a tightly regulated genetic cascade, as future reproductive potential relies heavily on the presence of primary sexual characteristics. Given the importance and conservation of sexual phenotypes, we might expect the genetic basis of sex determination itself to be highly conserved. However, this is not at all the case, with a remarkable diversity and turnover of both proximate and ultimate sex-determining mechanisms observed in many clades (Bachtrog et al. 2014).

Although sex determination can be environmentally determined by factors such as temperature or social cues, sex is often associated with sex chromosomes. Sex chromosomes were discovered by Nettie Stevens in 1905, who noted in mealworms that male cells carried one chromosome smaller than the rest, whereas female cells carried all equally sized chromosomes (Brush 1978; Stevens 1905; Abbott et al. 2017). Others had similar findings around the same time but still invoked environmental influences as the primary cause (Wilson 1906; Brush 1978), whereas Stevens stood firm on the interpretation that sex was genetically determined.

Following Stevens’ discovery, sex chromosomes have proved to exhibit remarkable interspecific and intraspecific diversity. It is clear that sex chromosomes have evolved independently numerous times and turnover from one system to another frequently (Bachtrog et al. 2014). This diversity has made it possible to test empirically the rich body of evolutionary theory that predicts each stage of sex chromosome evolution. As a result, numerous studies have identified convergent genomic patterns in independently formed sex chromosomes (Bachtrog et al. 2011; Bachtrog 2013), and speculated about the causes of the repeated origins of these unique regions of the genome (Wright et al. 2016). However, new data emerging from nonmodel sex chromosome systems provide interesting exceptions to long-standing theories on how sex chromosomes originate and evolve, and suggest a diversity to the process not previously acknowledged.

## Sex Chromosome Classification

For organisms that express sex in the diploid phase, there are two main types of sex chromosome systems. Stevens’ original discovery was that of an XX/XY system, where males are heterogametic with a Y chromosome and an X chromosome, and the Y is restricted to males. Others around that time (Wilson 1906) found a variant on this system, whereby males carry one fewer chromosome than females, called an XX/X0 system. There is also a converse system, female heterogamety, designated as ZW/ZZ, with the W chromosome associated with females. Cases in which females have one fewer chromosome than males are correspondingly called Z0/ZZ. XX/X0 and Z0/ZZ systems are often assumed to result from the loss of the Y or W chromosome, presumably in systems where sex is ultimately determined by a dosage-based gene on the X or Z chromosome (though that is not always the case; Kuroiwa et al. 2010).

Genetically determined sex can also occur in the haploid phase of life for some organisms, including mosses and algae, and the sex chromosomes in these cases are designated U and V (Bachtrog et al. 2011). In these systems all individuals are heterogametic in the diploid phase, carrying both a U chromosome and a V chromosome. In the haploid phase, individuals have either a U or V chromosome. More complicated schemes can also be found for many fungi, where multiallelic systems operate to define genetically distinct mating types and recombination only proceeds when two haploid genomes of different mating types meet (Nieuwenhuis and James 2016). As well, there are numerous other modes of genetic sex determination, including haplodiploidy (e.g., Hymenoptera, where males are haploid and females are diploid), polygenic systems, and others (outlined in Bachtrog et al. 2011). Here, we mostly restrict our review to the evolutionary forces affecting ZW and XY systems, but touch on insights that can be gained from other systems, like the mating type locus of fungi.

In addition to the different types, sex chromosomes can be heteromorphic, with some degree of genetic divergence, ranging from SNPs, inversions and/or deletions, between the sex chromosomes. Alternatively, sex chromosomes can be homomorphic, with relatively little divergence observed between the pairs. There is thus far no consensus for the point at which a sex chromosome pair is classified as heteromorphic or homomorphic, although many assessments of heteromorphy are based on whether chromosomal karyotypes are visibly different between females and males. The degree of divergence is not necessarily associated with sex chromosome age (Wright et al. 2016), with examples of young heteromorphic systems (Darolti et al. 2019) and old homomorphic systems (Stöck et al. 2011).

## The Origin of Sex Chromosomes

For sex to be genetically determined, a genetic variant must gain control over the sex-determination cascade, often referred to as a master sex-determining gene. This can occur through a point mutation in a gene, knocking out function or creating a new function (Kamiya et al. 2012; Myosho et al. 2012), gene duplication followed by neofunctionalization (Yoshimoto et al. 2008; Harkess et al. 2017), deletion (Smith et al. 2009), regulatory change (Herpin et al. 2010), and perhaps other ways yet undiscovered. The master sex-determining gene can act in a dominant fashion on the Y or W chromosome, where one copy is needed to determine maleness (on a Y chromosome) or femaleness (on a W chromosome). Alternatively, the master sex-determining locus can act in a dose-dependent manner on the X or Z chromosome, where two functional copies are needed for femaleness (on the X chromosome) or maleness (on the Z chromosome).

The classic model (Bull 1983; Charlesworth 1991) assumes that sex chromosomes arise from a pair of autosomes following the acquisition of the master sex-determining locus. Many sex chromosomes follow this model and descend from a pair of once homologous autosomes. This is clearly evident from the shared gene content observed as X–Y or Z–W orthologs seen in therian mammals (Lahn and Page 1999), *Silene* (Filatov 2005), fish (Kamiya et al. 2012; Natri et al. 2013), snakes (Vicoso et al. 2013), birds (Wright et al. 2012, 2014), and many others.

However, there is increasing evidence that the sex-limited chromosome in some systems arose independently and does not share a common ancestry with the X or Z. For example, B chromosomes, small, nonessential chromosomes that are often selfish in their transmission, act as the Y chromosome in *Rhinocola aceris* and *Cacopsylla peregrina* (Nokkala et al. 2000, 2003), as well as a W chromosome in some Lake Malawi cichlids (Clark and Kocher 2019). There is strong evidence that the W chromosome in Lepidoptera arose after the origin of the Z, possibly from a B chromosome (Fraïsse et al. 2017). In the case of the pillbug (*Armadillium vulgare*), the W chromosome arose from a Wolbachia feminizer that has been incorporated into the nuclear genome (Leclercq et al. 2016). This raises the intriguing possibility that cytoplasmic male sterility factors, common in both insects and plants, could present opportunities for the origin of nonhomologous W chromosomes when they are transferred to the nuclear genome.

### A Note about the Origin of Plant Sex Chromosomes

The classic model for sex chromosome evolution in plants is slightly different from that outlined above. Instead of a single locus initiating the development of one sex, the plant model requires two linked loci, one each for female and male sterility (Westergaard 1958; Charlesworth and Charlesworth 1978). This difference results from the fact that most sex chromosomes in plants originate in monoecious or hermaphroditic lineages where both sexes (referred to as genders in the botanical literature) are present in the same flower, or flowers of each sex are present on the same plant, whereas separate sexes predate the origin of most sex chromosome systems in animals. There is some evidence for the two-locus model in plants, including kiwi fruit (Akagi et al. 2019) and asparagus (Harkess et al. 2017). However, there is clear evidence that this is not the only route to sex chromosome evolution in plants. In *Mercurialis annua*, a system with homomorphic sex chromosomes, individuals sometimes exhibit intermediate states, producing some flowers of the opposite gender and suggesting that sterility in at least one gender is quantitative (Cossard and Pannell 2019) rather than controlled by a single sterility locus. Furthermore, evidence from wild strawberry (Tennessen et al. 2018), persimmon (Akagi et al. 2014), and the Salicaceae (willows and poplar, Müller et al. 2020; Almeida et al. 2019) suggest sex determination is single-locus in these species, rather than controlled by two linked sterility loci.

The prevalence of cytoplasmic male sterility factors in plants presents a particularly interesting possible role in sex determination. It is possible that the male sterility factor could become a W chromosome, as in the case of pillbugs described earlier (Leclercq et al. 2016). Alternatively, the male sterility resistance locus could conceivably become a Y chromosome (Willis J, personal communication).

## Selection against Recombination

After the acquisition of a master sex-determining gene, sex-linked regions may be small and can be associated with just a single nucleotide, as in the case of pufferfish, where a single missense SNP in the proto-Y chromosome is associated with male development (Kamiya et al. 2012). Though not universal (Wright et al. 2016), recombination suppression between the X and Y or Z and W is a recurrent phenomenon, leading to sex chromosome divergence and heteromorphy (Bachtrog et al. 2014). Classic models of heteromorphic sex chromosome formation suggest that the acquisition of sexually antagonistic alleles, which confer a fitness advantage in one sex but a cost in the other, near a sex-determining locus is a primary driver of recombination suppression (Fisher 1931; Rice 1987). In a male heterogametic system, linkage ensures that an allele that confers maleness is always coinherited with nearby alleles that confer benefits to males. This theory was inspired in part by the observation that many male color patterns in guppies are inherited through the patriline, consistent with Y linkage (Winge and Winge 1927).

Linkage evolves to resolve sexual conflict, as Y-linked male-benefit loci are no longer present in females and selected against. The role of sexual conflict in recombination suppression has been particularly challenging to test empirically, largely due to the difficulty in identifying the genomic location of sexually antagonistic alleles. A recent test of this theoretical step in the evolution of sex chromosomes in guppies found that the nonrecombining region has expanded independently in multiple populations where female preference for male color is stronger. Presumably, greater female preference produces greater levels of sexual conflict, therefore selecting for expansion of the nonrecombining region (Wright et al. 2017). The Y chromosome in this region shows low levels of divergence from the X (Wright et al. 2017; Darolti et al. 2019) despite the fact that recombination suppression is not quite complete (Winge and Winge 1922, 1927; Yamamoto 1975; Bergero et al. 2019), suggesting that recombinants are selected against in natural populations. Alternatively, the buildup of mutations on the Y chromosome may be faster than what rare recombination events between the X and Y can counter. However, Wright et al. (2017) did not map sexually antagonistic alleles directly, and this therefore remains an oblique test.

Importantly, recombination suppression has occurred in systems without sexes, which by definition lack sexual conflict (Branco et al. 2017; Sun et al. 2017), suggesting that sexual conflict may not underlie all cases of sex chromosome formation (Ironside 2010). Consistent with this, in systems where one sex is achiasmate (completely lacking recombination anywhere in the genome), the emergence of a nascent sex-determining factor leads to instantaneous recombination suppression along the entire length of the emergent sex chromosomes independent of linked sexually antagonistic loci (Wright et al. 2016), regardless of nearby sex-linked loci. Further review of the evolutionary pressures that drive recombination suppression can be found in Charlesworth (2017) and Ponnikas et al. (2018).

It is important to note that selection against recombination does not necessarily mean that recombination never occurs between the X and Y or Z and W, rather recombinant individuals are at a fitness disadvantage. This distinction is particularly important in studying nascent sex chromosome systems where recombination suppression is not complete, as X–Y or Z–W divergence may be observed even in the presence of occasional recombination if recombinant individuals are less fit. In these cases, genetic mapping of the nonrecombining region based on observed crossing-over events in a lab population will be less effective than population-based sequencing approaches that measure sex chromosome divergence. This is because the latter approach measures the net effects of both recombination and selection against recombinants.

## Mechanisms of Recombination Suppression

Selection against recombinants is expected to ultimately lead to mechanisms that that act to suppress recombination itself, of which several possibilities exist.

### Inversions

Chromosomal inversions spanning the sex-determining locus and other loci are often assumed to be the cause of recombination suppression, halting recombination for all the encompassed loci simultaneously (Charlesworth et al. 2005). Once recombination has been initially suppressed, additional inversion events can, in the same way, progressively extend the nonrecombining region of the sex chromosomes (Otto et al. 2011), resulting in distinct regions of different ages and different degrees of degeneration depending on the age of the inversion, often referred to as strata. Strata are generally defined as regions where genomic characteristics cluster into distinct groups. The oldest stratum typically represents the initial recombination suppression event and is characterized by the greatest accumulation of genetic divergence between the sex chromosomes. Younger evolutionary strata are characterized by a lesser degree of divergence. Consistent with this, strata were first observed in comparisons of divergence of X–Y orthologs in therian mammals which clustered into several clear categories spatially across the chromosome (Lahn and Page 1999). Many other sex chromosome systems have subsequently been shown to contain strata (Bergero et al. 2007; Wang et al. 2012; Roesti et al. 2013; Vicoso et al. 2013; Wright et al. 2014).

However, there is little direct evidence that inversions actually serve to halt recombination on sex chromosomes. A series of recent comparative genomic analyses in fungi have convincingly demonstrated that recombination suppression was the ancestral state, and inversions are a consequence, rather than the primary cause, of halted recombination (Grognet et al. 2014; Branco et al. 2017; Sun et al. 2017; Carpentier et al. 2019). Inversions are likely to follow recombination suppression by other means, as the loss of recombination leads to the loss of selection to maintain gene order. Detailed studies in many of the well-characterized sex chromosome systems have also challenged the notion of strict strata boundaries induced by inversions in favor of a more gradual and continuously evolving process resulting in the expansion of the nonrecombining regions (Iwase et al. 2003; Cotter et al. 2016; Campos et al. 2017; Wright et al. 2017; Li et al. 2019; Xu et al. 2019). This suggests that although the strata definition, regions with genomic characteristics which cluster spatially, is still useful, we might be better served to envision the boundaries between strata as fuzzy, rather than strictly discrete.

**Fig. 1.**
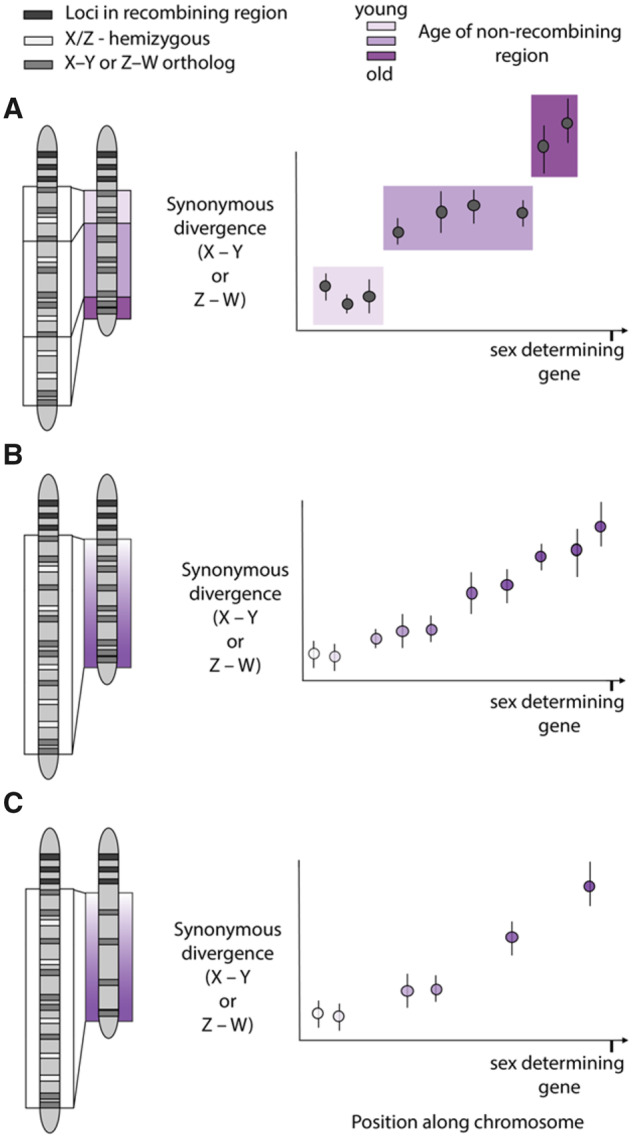
—Expected patterns of sex chromosome divergence following recombination suppression. (*A*) Stepwise progression, due to inversions or large shifts in recombination hotspots, results in large spatial blocks where the divergence between X–Y or Z–W orthologs is similar. (*B*) Progressive expansion (e.g., TE accumulation, methylation changes) results in a linear relationship between ortholog divergence across the range of the sex chromosome. (*C*) A potential problem of only sampling a few genes is that a stepwise pattern may be inferred, when it was truly progressive expansion. A similar pattern could happen if inversions, or other discrete changes, reinforce pre-existing recombination suppression soon after it is established.

Furthermore, nascent sex chromosomes show heterogeneous divergence rates between the sex chromosomes (Bergero et al. 2013; Natri et al. 2013; Reichwald et al. 2015; Almeida et al. 2019) and are inconsistent with a single inversion event and therefore suggest that recombination suppression evolves by a more progressive mechanism, and may be incomplete initially. Discrete and progressive recombination suppression will leave distinct patterns in divergence between the sex chromosomes, but it is worth noting that progressive expansion of the nonrecombining region with sparse sampling of X–Y orthologs could give a false signal of strata (fig. 1).

### Transposable Elements

Transposable elements (TEs) are selfish genetic sequences capable of replicating and inserting themselves throughout the genome. Although often assumed to accumulate following recombination suppression, the insertion of TEs near the sex-determining locus can also act to suppress recombination by creating divergence between sex chromosomes. This would invoke host mechanisms to silence TEs, resulting in suppressed recombination at hotspots adjacent to TE insertions (Kent et al. 2017). Once a lack of recombination is established, there is less selection against the insertion of more TEs, leading to their accumulation. In recently established regions of suppressed recombination in both mammals and birds, TEs are found at boundaries of recombining and nonrecombining regions, suggestive of their causal role (Iwase et al. 2003; Xu et al. 2019). Nascent sex chromosome systems also show accumulation of TEs, such as in spinach and papaya (Na et al. 2014; Li et al. 2019). Furthermore, TEs can promote ectopic recombination, facilitating genomic rearrangement to further suppress recombination (Bonchev and Willi 2018). These findings suggest that TEs, and other repetitive sequences (Reichwald et al. 2015), may play a critical role in the early stages of recombination suppression. Given the ability of TEs to shuffle genes and alter expression patterns, it is possible that TEs could simultaneously promote turnover of sex chromosomes and sex-determining genes, while also initiating recombination suppression (Ponnikas et al. 2018).

### Recombination Modifiers and Epigenetic Changes

Sex chromosomes with persistent homomorphy, even in the presence of recombination suppression (Brelsford et al. 2016; Furman and Evans 2018; Veltsos et al. 2019) may suggest that some mechanisms of recombination suppression are reversible. For many eukaryotes, crossover events are concentrated into hotspots (Baker et al. 2017). In some vertebrates, this is driven by PRDM9, a zinc finger protein that binds to specific DNA motifs and subsequently recruits the recombination machinery. These binding motifs change rapidly and are preferentially extinguished in favor of alleles that recombine less (Myers et al. 2010), and any selection against their reestablishment on a sex chromosome could promote a recombination coldspot.

Alternatively, DNA methylation and histone modifications are known to be involved in regulating chromatin structure and gene expression, but how these two epigenetic processes interact is complex and context dependent (for review see Cedar and Bergman 2009). Although there is no firm rule governing the relationship between DNA methylation and histone modifications, hypermethylation of DNA, and trimethylation of histone H3 at lysine 9 (H3K9Me3) is commonly associated with transcriptionally repressed chromatin, and can reduce recombination across large regions of the chromosomes. Although direct evidence linking epigenetics to recombination suppression is lacking, high levels of DNA methylation were associated with nonrecombining regions of sex chromosomes in sticklebacks and papaya (Zhang et al. 2008; Metzger and Schulte 2018), suggesting DNA methylation may play a role in recombination suppression.

Overall, the relationship between epigenetic modifications and sex chromosome evolution is not well understood and is often overlooked, but some propose that DNA methylation could play an integral role in the formation of heteromorphic sex chromosomes (Gorelick 2003; see box 1

Box 1 The Role of Epigenetics in the Evolution of Heteromorphic Sex Chromosomes

Epigenetic mechanisms such as DNA methylation, the addition of a methyl group (CH3) to the 5′-carbon of a cytosine nucleotide (5-methylcytosine), and histone modifications, the posttranslational modification of a histone “tail,” are known to be involved in regulating chromatin structure and gene expression activity. However, the relationship between epigenetic modifications and recombination suppression and divergence of sex chromosomes is not well established. Gorelick (2003) proposed a compelling theory intertwining epigenetic modification and Müller’s ratchet. First, a potential sex-determining locus would be differentially methylated between homologous chromosomes with the more highly methylated of the two homologous chromosomes eventually becoming the heteromorphic (Y or W) sex chromosome. This differential methylation could then result in the expression and development of sex-specific characteristics in the heterogametic sex, and create recombination reducing chromatin modifications. Second, methylated cytosines are hypermutable and can deaminate to become thymines at a faster rate compared with unmethylated cytosines. Thus, this locally differentiated methylation would accelerate Müller’s ratchet by increasing the mutation rate and accelerate the divergence of sex chromosomes as methylated CpG sites degrade to TpG sites (Sved and Bird 1990; Holliday and Grigg 1993).

Making predictions about DNA methylation status of the sex-determining region at the initiation of sex chromosome divergence is not straightforward, as regulation of gene expression by DNA methylation can be complex (Jaenisch and Bird 2003). For example, hypermethylation of promoter regions is associated with a transcriptionally repressed state. In contrast, hypermethylation within gene bodies is associated with active transcription. DNA methylation can also regulate the activity of regulatory elements that can be located several megabases away from the genes that they influence, and could have conflicting effects on a gene depending on whether these elements are repressors or enhancers (Jaenisch and Bird 2003). The requirement of acquired DNA methylation patterns may also be problematic. In some organisms, DNA methylation patterns are erased during development (e.g., mammals), whereas in others (e.g., zebrafish) DNA methylation levels are maintained through fertilization and development. It is possible that a potential role of epigenetic processes in the evolution of sex chromosomes is more plausible in species that lack DNA methylation reprogramming, however, the dynamics DNA methylation reprogramming and reestablishment during development are not well understood, making a generalization about the effects of DNA methylation reprogramming on the heritability of acquired DNA methylation patterns difficult.

Taken together, these ideas make testing Gorelick’s hypothesis challenging. Higher levels of methylation are essential in this theory because of their effects on chromatin structure, recombination, and mutation rates. In cases where the sex-determining gene is expressed in the heterogametic sex, the conventional regulation of gene expression through promoter methylation would not result in the necessary pattern of sex-biased methylation required of Gorelick’s hypothesis. In this scenario, one might expect the hypermethylation of the sex-determining region to only occur within gene bodies. Alternatively, in a sex-determination system in which the effect of the sex-determining gene is associated with decreased expression in the heterogametic sex (e.g., dmrt1 in birds), hypermethylation of the promoter region in the heterogametic sex would be consistent with Gorelick’s theory.

As well, the expected DNA methylation pattern of the sex-determining locus depends on the amount of divergence between the sex chromosomes along with the methodology used to detect DNA methylation. Many reference genomes are sequenced and assembled from the DNA of the homogametic sex (XX female or ZZ males). Thus, if methylated cytosines in the nonrecombining region accelerate Müller’s ratchet, then the frequency of CpG sites in the nonrecombining region of the heteromorphic chromosome would be depleted due to the conversion of methylated cytosines to thymines. Consequently, when analyzing whole-genome bisulfite sequencing (WGBS) data, the nonrecombining region between diverged heteromorphic sex chromosomes would appear to be more highly methylated in the homogametic sex compared with the heterogametic sex. Although this pattern might appear to contrast the initial prediction of hypermethylation in the heterogametic sex, it is instead consistent with what might be expected using a bisulfite sequencing approach in a more derived sex chromosome system, as has been previously observed (Metzger and Schulte 2018).

Advances in modern sequencing technologies provide powerful new approaches to begin to address some of the more outstanding questions linking epigenetic processes and sex chromosome evolution in nonmodel organisms. The use of techniques providing chromosomal architecture (e.g., ATAC-seq, ChIP-Seq) around a sex-determining locus may provide functional support for an epigenetically driven mechanism when coupled with DNA methylation or histone modification data.).

As well, for homomorphic sex chromosomes, which contain largely the same genetic content, methylation differences between the sexes could allow for differential expression of these shared genes.

DNA methylation can also interact with other influencers on sex chromosome recombination rates. This could create an environment conducive to further differentiation of the sex chromosomes. As discussed earlier, TEs may play an integral role in the early stages of sex chromosome formation, and their repression by DNA methylation changes could set off a cascade of mutation accumulation and reduced gene expression for genes on the sex-limited Y or W chromosome (Slotkin and Martienssen 2007; Zamudio et al. 2015).

## Intraspecific Variation in Sex-Linked Regions

Curiously, there are many reports of intraspecific variation in the nonrecombining region in vertebrates (table 1), even when not accounting for fusions that create neo-sex chromosomes. Karyotype studies demonstrated substantial variation in sex chromosome differentiation within species (Green 1988; Bellafronte et al. 2009; Shibaike et al. 2009), as have some RAD-Seq surveys (Wilson et al. 2014; Utsunomia et al. 2017). However, whole-genome sequencing studies tend to focus on relatively few samples, assumed to be representative of the species, although there are notable exceptions (Reichwald et al. 2015). Given the fact that some species show variation in sex chromosome system across individuals and populations—notably in various *Rana* species (Wright and Richards 1983; Sumida and Nishioka 1994)—it seems likely that intraspecific diversity within sex chromosome systems can be high, particularly for young sex chromosomes, or the leading front of older sex chromosomes, where fixation has not yet had sufficient time to occur.

**Table 1:** **Genomic Evidence of Intraspecific Diversity in the PAR-Sex Chromosome Boundary in Vertebrates** ^a^

Group	Species	Type	References
Fish	*Nothobranchius furzeri*	Within the small nonrecombining region, there is variation across lab populations/strains in linkage between SNPs and sex-determining region. In addition, there is structural variation on the sex chromosome across populations.	Reichwald et al. (2015)
	*Poecilia reticulata*	Variation across populations in physical size of the Y chromosome; extent of Y differentiation and extent of nonrecombining regions.	Darolti et al. (2019); Morris et al. (2018); Nanda et al. (2014); Wright et al. (2017)
	*Characidium gomesi*	Variation across populations in W-linked RAD markers.	Utsunomia et al. (2017)
	*Danio rerio*	Sex chromosome in wild strains not present in domestics.	Anderson et al. (2012); Wilson et al. (2014)
	*Apareiodon ibitiensis*	Variation across populations in sex-linked satellite DNA accumulation.	Bellafronte et al. (2009)
Amphibians	*Leiopelma hochstetteri*	C-banding patterns on the W chromosome vary substantially across populations.	Green (1988)
	*Rana temporaria*	Northern populations show greater X–Y F_ST_ than southern populations.	Rodrigues et al. (2014); Rodrigues et al. (2016)
	*Aneides ferreus*	Structural variation in ZW system.	Kezer and Sessions (1979)
Reptiles	*Zootoca vivipara*	Structural and heterochromatin variation between viviparous and oviparous populations.	Odierna et al. (1998)
	*Gekko japonicus*	Variation in degree of sex chromosome differentiation across populations.	Yoshida and Itoh (1974); Chen et al. (1986); Gamble (2010)
	*Cyrtodactylus pubisulcus*	Variation in degree of sex chromosome differentiation across populations.	Ota et al. (1992)
Mammals	*Mus musculus*	Shifted PAR boundaries between subspecies.	White et al. (2012)

a Does not include cases of neosex chromosome fusions.

In many ways, it makes inherent sense that there might be intraspecific variation in the degree of sex chromosome differentiation. Even if sex chromosome differentiation is at least partly explained through adaptive processes, for example, sexual conflict (Fisher 1931; Bull 1983; Rice 1987; Charlesworth 1991), it takes time for these variants to fix within a species, leading to periods of polymorphism. In addition, it is entirely possible that the extent of sexual conflict differs across populations that experience different behavioral ecologies, leading to variation in the level of sex chromosome differentiation. For young, homomorphic sex chromosomes there may simply not have been enough time for a feature that suppresses recombination to fix across a species range. Comparative studies seeking to test various theories of sex chromosome formation have tended to focus on interspecific data (Pokorná and Kratochvíl 2009; Pennell et al. 2018), seeking to harness the remarkable diversity observed in many broad clades. But it may be that comparing across populations within species is more powerful for testing theories of sex chromosome evolution than comparisons across species, as there may be fewer other factors to consider given the more recent shared ancestor and ongoing gene flow.

The variation within taxa can provide compelling evidence as to what may be causing sex chromosome recombination suppression. Chromosomal rearrangements like inversions are rare events that take time to fix within a species, particularly if sexual conflict is not involved and they are largely neutral in their fitness effects (Ironside 2010; Branco et al. 2017). Thus, comparisons among populations could reveal a segregating inversion, capable of expanding the boundaries of recombination suppression (Reichwald et al. 2015). Alternatively, recombination patterns between populations are known to differ (Kong et al. 2010), and could lead to variability in the degree of divergence between sex chromosomes without the need for inversions (Wright et al. 2016). By assessing whether differences in the recombination landscapes among populations align with differences in sequence divergence, it may be possible to deduce that shifting recombination coldspots might be responsible for establishing recombination suppression. Overall, acknowledging and utilizing this variation, where possible, can help exclude seemingly obvious candidates of recombination suppression (Reichwald et al. 2015; Sun et al. 2017).

## Reevaluating the Necessity of Chromosomal Dosage Compensation

Once recombination is permanently halted, multiple evolutionary processes act to erode the sex-limited chromosome through mutation accumulation and gene loss (reviewed in Bachtrog 2013; Bachtrog et al. 2019). As a consequence, X- or Z-linked genes become increasingly monoallelic in the heterogametic sex, whereas the homogametic sex retains two functional copies. For many loci, a reduction in gene dose correlates with a reduction in gene activity and expression, which can be reflected in the level of protein abundance (Malone et al. 2012). Moreover, in complex interconnected networks that integrate both sex-linked and autosomal loci, changes in gene dose can disrupt the balanced protein ratios required for proper network functioning (Birchler and Veitia 2010). The effects of gene dose differences for sex-linked loci can thus resonate across the entire genome and negatively impact fitness in the heterogametic sex.

The consequences of Y or W chromosome degeneration are often hypothesized to create the need for the evolution of dosage compensation mechanisms that would restore expression to the ancestral, balanced state found before sex chromosome decay and gene loss (Ohno 1967). Dosage compensation was originally thought to occur across the entirety of the X or Z chromosome, evolving primarily in response to selection for hyperexpression in the heterogametic sex in order to achieve parity between the sex chromosomes and the autosomes (Ohno 1967). Transcription rates, however, can be strongly correlated between the two sexes, and thus compensation for dosage imbalance in the heterogametic sex may cause a detrimental overexpression of sex-linked loci in the homogametic sex (Wright and Mank 2012). As a result, a secondary, sex-specific process may evolve to restore optimal, balanced expression in the homogametic sex and equalize transcription between males and females (Ohno 1967). In line with this model, early studies in model organisms discovered complex, independently evolved strategies of regulating chromosome-wide transcriptional rates in order to mitigate the effects of sex chromosome differentiation (Mank 2009).

### Gene-by-Gene Dosage Compensation

Initial findings prompted the expectation that dosage compensation is an indispensable, universal process that evolves alongside Y or W chromosome degeneration (Ohno 1967). However, studies across a broader range of taxa have refined this view, and suggest that complete dosage compensation is the exception rather than the rule. Although few other systems exhibit global or near complete sex chromosome dosage compensation (Smith et al. 2014; Jiang et al. 2015; Pal and Vicoso 2015; Walters et al. 2015; Rose et al. 2016; Gopinath et al. 2017; Gu et al. 2017; Huylmans et al. 2017; Rupp et al. 2017; Davis et al. 2018; Darolti et al. 2019), incomplete dosage compensation, with no associated deleterious effects, is present in many species with sex chromosomes at different stages of divergence, including in birds (Itoh et al. 2007), snakes (Vicoso et al. 2013), many fish (Leder et al. 2010; Chen et al. 2014; Hale et al. 2018), frogs (Malcom et al. 2014), and plants (Muyle et al. 2012; Hough et al. 2014). Regulation of gene dose in these species operates locally, on a gene-by-gene basis for haploinsufficient genes, however, average expression on the sex chromosomes is significantly reduced compared with that on the autosomes in the heterogametic sex or compared with the sex chromosome in the homogametic sex (Mank 2009).

These striking results raise the questions of when and why sex chromosome dosage compensation evolves. Interactions between gene dosage and transcriptional output are not always linear and as a result not all sex-linked genes are similarly sensitive to dose (Malone et al. 2012). In addition, pre-existing expression buffering systems can act on single-copy genes to mitigate the effects of aneuploidy (Stenberg and Larsson 2011). As a result, only a minority of loci are thought to be dosage sensitive.

Specific gene properties may play a role in the evolution of dosage compensation as well. In particular, lowly expressed genes tend to exhibit fewer dosage effects, perhaps due to the fact that the transcriptional process is less saturated at lower expression levels (Harrison et al. 2012). Moreover, ohnologs, gene duplicates retained for long periods of time after whole-genome duplications are thought to be particularly sensitive to gene dose. This is because their retention within the genome is required in order to maintain dosage balance. Sex-linked ohnologs have been found to be associated with dosage-sensitive functions and preferentially compensated (Zimmer et al. 2016). In addition, like ohnologs, drift could play a role in establishing dosage compensation-like patterns, and take an extended period of time to become established (Gout and Lynch 2015), as the sex-shared copy drifts to higher expression levels while the sex-specific copy progressively degrades.

With some exceptions (Smith et al. 2014; Gopinath et al. 2017; Huylmans et al. 2019), global sex chromosome dosage compensation has been predominantly observed in XY systems, however, this tendency is based on relatively few examples and there is a clear need for greater sampling. Thus, rates of evolution for dosage compensation mechanisms may vary between male- and female-heterogametic systems (Mullon et al. 2015). This variation could be in part driven by the generally higher rates of mutation in males (Wilson Sayres and Makova 2011) that would cause Y chromosomes to accumulate mutations and degenerate faster than W chromosomes. In theory, a slower rate of genetic decay would weaken selection for chromosome-wide dosage compensation in ZW systems. In addition, reproductive variance is often greater in males, reducing the effective population size, and implicitly the rate of adaptation, of Z chromosomes relative to X chromosomes (Mank et al. 2010; Wright et al. 2015). As such, these forces would lead to accelerated rates of evolution of dosage compensation in XY systems compared with ZW systems (Mullon et al. 2015). It is important to point out that the evolution of a complete system of sex chromosome dosage compensation would reduce purifying selection on the Y chromosome to maintain expression for dosage-sensitive genes, thus resulting in a positive feedback loop and accelerating Y chromosome regulatory decay.

## The Sex Chromosome Cycle

**Fig. 2.**
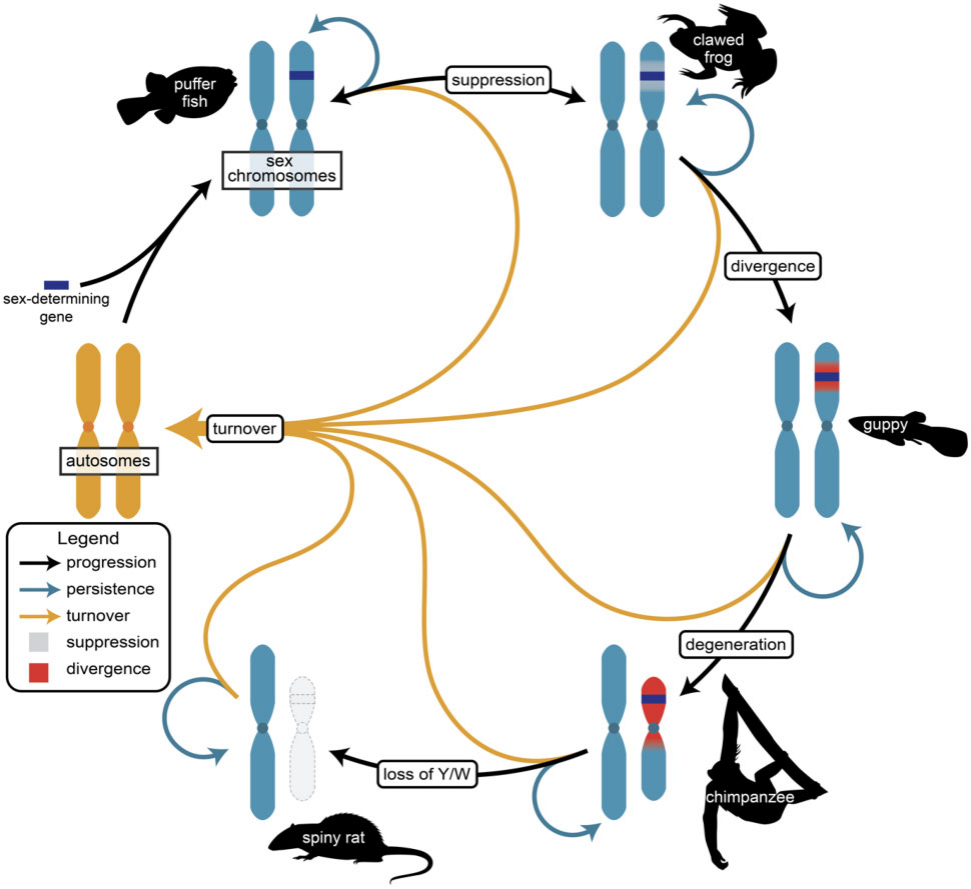
—The cycle of sex chromosome evolution. A new master sex-determining locus arises on an autosome (gold chromosomes, left side), leading to sex chromosome formation (blue chromosomes, starting top left), but sex chromosome evolution is not a simple progression of accumulating divergence. After establishment and at any stage of evolution, sex chromosomes can persist at the current stage (blue arrows), progress in establishing larger areas of recombination suppression (gray region, top right chromosomes) and divergence (red region, right side chromosomes), or turnover (gold arrows) with either a new sex-determining gene evolving or moving the sex-determining gene to a new location in the genome. Each stage here is highlighted by a representative taxon that currently possesses that sex chromosome state.

The theory of sex chromosome evolution implies a successive expansion and decay of the region surrounding the sex-determining locus, with an inevitable progression from homomorphic to heteromorphic sex chromosomes. For this to happen, the location of the sex chromosome within the genome must remain stable for long periods of time. However, broad comparative studies reveal that sex chromosomes are often ephemeral (Bachtrog et al. 2014; The Tree of Sex Consortium 2014), frequently shifting between chromosomes, and that sex chromosome evolution may be more cyclical than linear (fig. 2). Comparative genomics surveys in multiple closely related species have revealed that some clades are characterized by extensive turnover, including lizards (Gamble et al. 2015), fish (Myosho et al. 2015), amphibians (Jeffries et al. 2018; Cauret et al. 2019), insects (Vicoso and Bachtrog 2015), and plants (Balounova et al. 2019), all of which are characterized by frequent changes in the location of the sex chromosomes.

Sex chromosome turnover can occur when the existing master sex-determining gene physically moves onto an autosome and retains its control over sex determination. Although this is the most straight-forward transition theoretically, it is also one of the most difficult to demonstrate, as it requires knowledge of the sex-determining gene in multiple species. Nevertheless, recent work has shown that this has occurred in Northern Pike (Pan et al. 2019), independently across lineages of Atlantic salmon (Lubieniecki et al. 2015), and wild strawberry (Tennessen et al. 2018). Interestingly, the process of moving genes often takes surrounding sequence with it, which can link the sex-determining locus with genes that have sex-specific effects (Tennessen et al. 2018).

Turnover can also occur when a new master sex-determining gene arises de novo on an autosome (called nonhomologous turnover). The emergence of a new locus controlling the sex-determination pathway can have very different consequences depending upon how it interacts with the previous sex-determination system. When the new locus is dominant to the previous sex-determining system there is an instant turnover in which chromosome is acting as the sex chromosome (e.g., boas and pythons; Gamble et al. 2017).

In some cases, the emergence of a new sex-determining locus leads to a transition between XY and ZW systems, as has occurred in snakes and amphibians. Although most snakes share the same ancestral ZW chromosomes, with varying degrees of W degeneration, multiple pythons were found to have transitioned to XY systems (Gamble et al. 2017; Augstenová et al. 2018). Although one of the new XY systems shares gene content with the ancestral ZW chromosomes, the other new XY does not, suggesting that an autosome is now the sex chromosome (Augstenová et al. 2018). Amphibians also exhibit numerous transitions between ZW and XY systems (Roco et al. 2015; Jeffries et al. 2018; Cauret et al. 2019). It is worth noting that when turnover occurs between male and female heterogamety, there may be a period of transition where both XY and ZW sex chromosomes can coexist within a lineage (Roco et al. 2015; Gammerdinger and Kocher 2018) and even play out in hierarchical fashions (e.g., the YWZ of *Xenopus tropicalis*, Roco et al. [2015] or *Astatotilapia burtoni*, Roberts et al. [2016]).

When a new sex-determining gene arises on the previously existing sex-determining chromosome it is called homologous turnover. Although this does not act to change which chromosome is the sex chromosome, it has important implications for turnover between XY and ZW determination systems.

The theory behind how and why these turnovers happen was recently reviewed by Vicoso (2019) and Palmer et al. (2019), and assumes sexual conflict as a driver (van Doorn and Kirkpatrick 2007, 2010). However, the role of sexual conflict in turnover has been difficult to test empirically, in large part due to the difficulty in identifying sexually antagonistic alleles within the genome. Other models incorporate genetic drift (Bull and Charnov 1977; Veller et al. 2017), and mutation accumulation (Blaser et al. 2013, 2014).

### Stability of Sex Chromosomes

The stable, heterogametic sex chromosomes in some lineages, notably mammals and birds, were recently thought to be the result of an evolutionary trap; the sex-limited Y or W contains many genes with sex-specific effects, the loss of which would be detrimental to the heterogametic sex (Bull and Charnov 1977; Bull 1983; Pokorná and Kratochvíl 2009). However, recent work has shown that even in the XY system of mammals, thought to be one of the most stable, genes can move from the Y chromosome to the autosomes (Hughes et al. 2015), thereby permitting Y chromosome loss without fitness costs to the heterogametic sex and resulting in XO sex chromosomes, as observed in the Ryukyu spiny rat (Kuroiwa et al. 2010). Alternatively, sex chromosomes can be lost if the sex-determining locus no longer determines sex, as when genetic sex-determination transitions to an entirely environmental sex-determination system. Such transitions have recently been shown to occur in as little as one generation in bearded dragons (Holleley et al. 2015).

More broadly, comparative tests have shown that although transitions are common in homomorphic sex chromosomes (Muyle et al. 2012; Gamble et al. 2015; Myosho et al. 2015; Jeffries et al. 2018; Cauret et al. 2019), sex chromosome heteromorphy in general does not act as a brake against transitions (Pennell et al. 2018). There are also specific cases of turnover in highly heteromorphic systems with sex chromosomes reverting to autosomes (Vicoso and Bachtrog 2013). It therefore remains unclear why some sex chromosome systems persist for extensive periods of evolutionary time, whereas others are ephemeral (Vicoso 2019).

Sex chromosome turnover may ultimately be limited by the number of genes that can act as master sex-determining loci. A handful of genes with known sex-determination functions have been shown repeatedly to emerge as master sex-determining loci in animals, suggesting that there may be a core set of genes that can control sex determination (Marshall Graves and Peichel 2010; Bachtrog et al. 2014; Herpin and Schartl 2015). Though there may be some ascertainment bias whereby researchers are looking for known genes, resulting in an unfair assessment of the diversity of potential genes involved in sex determination, there are a number of cases involving unexpected candidates being found, such as growth factors and immune-related genes (Myosho et al. 2012; Yano et al. 2012). However, given the prevalence of gene duplication and movement, this does not necessarily limit the genomic location of sex chromosomes. Although sex chromosomes in some systems may share synteny (Furman and Evans 2016; Böhne et al. 2019), it is clear that synteny is not always a limitation on the genomic regions that can become sex chromosomes (Vicoso and Bachtrog 2015; Gammerdinger and Kocher 2018; Jeffries et al. 2018; Cauret et al. 2019; Meisel et al. 2019). These studies suggest that the genetic architecture of sex determination is dispersed throughout the genome.

## Concluding Remarks

Recent progress on sex chromosome evolution has in some cases supported long-standing theory, and in many others revealed that there is no single narrative for how these regions form and evolve. It is undisputed that sex chromosomes show convergent genomic signatures, suggesting broader trends in their formation. However, the diversity of sex chromosomes reveals a remarkable number of exceptions and therefore a parallel diversity of underlying mechanisms. This diversity suggests that the rules of sex chromosome evolution are variable, and not applicable to every species. The most informative systems moving forward may be those exhibiting the most variation in divergence or turnover, as these allow for comparisons to tease apart cause and effect. Furthermore, studies of young sex chromosomes are likely to reveal more about the formative processes, though these are also the most difficult to study given that divergence between the sex chromosomes is slight. Finally, recent work has shown that sex chromosome evolution can occur rapidly, making population-based approaches useful for understanding the mechanisms and patterns of early sex chromosome evolution. 
